# Sweet and umami TAS1R receptors: from molecular recognition to physiological function

**DOI:** 10.1093/chemse/bjag010

**Published:** 2026-03-19

**Authors:** Clémence Cornut, Christine Belloir, Adeline Karolkowski, Maxence Lalis, Sandrine Chometton, Sébastien Fiorucci, Jérémie Topin, Loïc Briand

**Affiliations:** Université Bourgogne Europe, Institut Agro, CNRS, INRAE, UMR CSGA, 21000 Dijon, France; Université Bourgogne Europe, Institut Agro, CNRS, INRAE, UMR CSGA, 21000 Dijon, France; Université Bourgogne Europe, Institut Agro, CNRS, INRAE, UMR CSGA, 21000 Dijon, France; Institut de Chimie de Nice UMR7272, CNRS, Université Côte d’Azur, 06108 Nice, France; Université Bourgogne Europe, Institut Agro, CNRS, INRAE, UMR CSGA, 21000 Dijon, France; Institut de Chimie de Nice UMR7272, CNRS, Université Côte d’Azur, 06108 Nice, France; Institut de Chimie de Nice UMR7272, CNRS, Université Côte d’Azur, 06108 Nice, France; Université Bourgogne Europe, Institut Agro, CNRS, INRAE, UMR CSGA, 21000 Dijon, France

**Keywords:** allostery, G protein-coupled receptors, nutrient sensing, structure–function, TAS1R2/TAS1R3, TAS1R1/TAS1R3

## Abstract

The detection of sweet and umami tastants is mediated by 2 heterodimeric G protein-coupled receptors, TAS1R2/TAS1R3 and TAS1R1/TAS1R3, respectively. Sweet taste provides input related to the carbohydrate-derived energy content of ingested food, whereas the physiological role of umami taste by detecting free L-amino acids is to signal the presence of protein-rich foods. In addition to being expressed in the oral cavity, TAS1R receptors are expressed in numerous extraoral tissues and organs, including the gut, where their physiological roles are not yet fully understood. In this review, we present an overview of the current knowledge on these taste receptors since their discovery in the early 2000s. We summarize the structure–function analyses, evolution, and expression of TAS1R genes and describe the molecular basis for the recognition of sweet and umami tastants. Together, these insights provide a comprehensive understanding of how TAS1R receptors contribute to nutrient detection and metabolic regulation both in taste perception and beyond.

## 1. Introduction

Taste is among the most important senses for determining food choice and regulating food intake among the human species. Five fundamental taste qualities, namely, sweet, salty, sour, bitter, and umami, are generally accepted. Before ingestion, taste provides us with information about the presence of carbohydrates, protein-rich foods, electrolytes, or potentially harmful compounds in food. The detection of sweet-tasting compounds enables us to identify foods with a high caloric content that can be directly assimilated, such as carbohydrates, whereas umami taste allows us to detect protein-rich foods marked by the presence of free amino acids. Sapid compounds dissolved in saliva are detected through the stimulation of specific receptors found in the mouth by numerous taste buds located mainly on the surface of the tongue. The detection of sweet and umami substances is mediated by class C G protein-coupled receptors (GPCRs) belonging to the taste receptor type 1 (TAS1R) family (Ahmad and Dalziel 2020). However, comparative genomic analyses have revealed that the TAS1R family is far more complex and heterogeneous in fish, with a larger and diversified repertoire of TAS1R genes, of which only a subset has been conserved and functions in higher vertebrates, including mammals (Itoigawa et al. 2024; Nishihara et al. 2024).

Sweet-tasting compounds are detected by a heterodimeric receptor made of the obligatory assembly of 2 subunits named TAS1R2 and TAS1R3. The 2 subunits, TAS1R1 and TAS1R2, were discovered in the early 2000s by searching for mRNAs preferentially expressed in mouse taste buds and genetically linked to the *Sac* locus, which was associated with sweet-taste sensitivity. They were initially named TR1 and TR2, respectively (Hoon et al. 1999). The discovery of the TAS1R3 subunit was achieved by independent research groups at around the same time (Bachmanov et al. 2001; Kitagawa et al. 2001; Max et al. 2001; Montmayeur et al. 2001; Nelson et al. 2001; Sainz et al. 2001). When coexpressed in cultured cells, TAS1R2 and TAS1R3 were shown to detect all the sweet-tasting compounds perceived by humans, including sugars, natural and synthetic sweeteners, and plant sweet-tasting proteins (Nelson et al. 2001; Li et al. 2002). Mice lacking TAS1R2 or TAS1R3 lose sensitivity to sugars and artificial sweeteners. However, the responses to glucose-containing sugars are not completely eliminated, suggesting that TAS1R3­independent mechanisms probably exist for the detection of this nutrient (Damak et al. 2003).

Umami taste was identified as a distinct taste in 1908 by a chemistry professor at the Imperial University of Tokyo, Kikunae Ikeda. He isolated L-glutamate (L-Glu) from the seaweed *Laminaria japonica* and was the first to describe its unique taste properties, which were distinct from those of the 4 other primary taste qualities. He named this taste umami, a word derived from the Japanese adjective umai meaning delicious (Ikeda 2002). Two other umami compounds were subsequently isolated: inosine-5′-monophosphate (IMP) from dried bonito (Kodama 1913) and guanosine-5′-monophosphate (GMP) from dried shiitake mushrooms (Kuninaka 1964). The 1960s were notable for the discovery that IMP and GMP not only cause umami taste themselves, but also significantly enhance the umami sensation induced by L-Glu (Kuninaka 1960; Kuninaka 1964; Yamaguchi 1991). This synergy is the most unique hallmark of umami taste. In the 1980s and 1990s, taste responses to umami tastants were investigated in humans and several animal species using mainly electrophysiology and behavioral experiments. Comparative studies revealed widely divergent taste preferences for L-amino acids from one species to another. For instance, in humans, only L-Glu and L-aspartate (L-Asp) generate umami taste, while rodents are able to perceive a wider range of L-amino acids (Li et al. 2002; Nelson et al. 2002). The identification of heterodimeric TAS1R1/TAS1R3 as a taste receptor specifically involved in the detection of umami molecules was a major discovery in the early 2000s (Nelson et al. 2002). Although TAS1R1/TAS1R3 appears to be the validated umami taste receptor, other candidate umami taste receptors have been proposed, including truncated variant of the metabotropic glutamate receptors (mGluRs), mGluR1 and mGluR4, which are expressed in taste cells and display reduced sensitivity compared with their full-length counterparts making them compatible with taste function (Chaudhari et al. 2000; San Gabriel et al. 2005; Yasumatsu et al. 2012). More recently, the class A GPCR GPR91 has been proposed to function as a gustatory receptor involved in the detection of the umami taste of succinate (Kitajima et al. 2025).

This review aimed to provide a comprehensive overview of the molecular diversity of compounds that induce sweet and umami flavors, from natural to synthetic sweeteners and potent umami compounds. We detail the current knowledge on the structural organization of TAS1R receptors, with a particular focus on their binding pockets and key amino acid residues involved in ligand recognition and selectivity. Finally, we discuss recent advances in structural biology, highlighting the first experimental structures of the sweet taste receptor that provide unprecedented insights into its activation mechanisms and ligand-binding modes.

## 2. Sweet-tasting compounds

**Figure 1:**
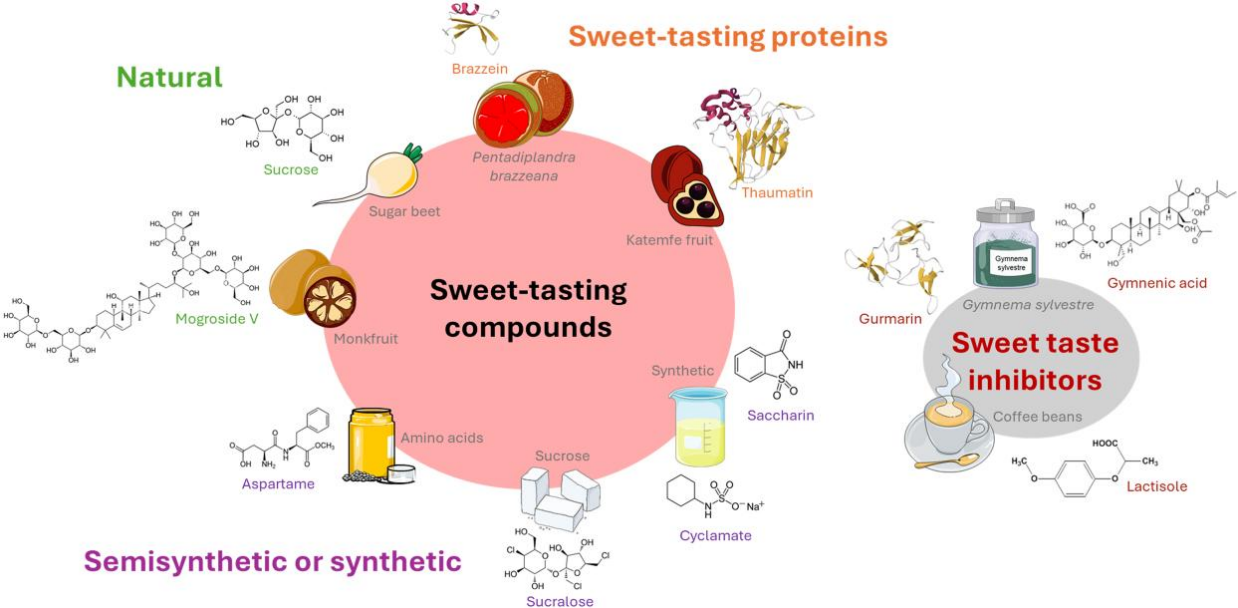
Sweet-tasting compounds and sweet taste inhibitors identified in certain food products. The chemical structure of each compound is shown, except for the sweet-tasting proteins brazzein and thaumatin, and the sweet taste-suppressing polypeptide gurmarin, which are composed of 54, 207, and 35 amino acid residues, respectively, and are represented by their 3D structures (PDB IDs: 1BRZ, 2VHR, and 5OLL, respectively). The α-helices and β-sheets are represented in red and yellow, respectively.

A diverse array of compounds elicits sweet taste perception through the activation of the TAS1R2/TAS1R3 sweet taste receptor. In addition, sweetness enhancers or inhibitors have been identified (Fig. 1). The sweetness of a compound can be evaluated using complementary methods based on sensory analysis and cellular-based assays. The sweetness potency of a substance is determined by comparing its perceived sweetness intensity to that of a reference, typically sucrose, at equivalent concentrations. This approach allows the sweetness of a compound to be measured directly in comparison with conventional sweet-tasting compounds. The detection threshold method assesses the minimum concentration at which a compound can be perceived as sweet by human subjects, thus reflecting its sensory sensitivity. At the molecular level, EC_50_ value measurements quantify the concentration required to activate 50% of the human sweet taste receptor response in cell-based assays, which can be extrapolated to sweetness intensity.

### 2.1 Natural sugars

Natural sugars encompass a wide range of carbohydrates, from simple monosaccharides to complex polysaccharides, which contribute to the perception of sweetness in food. Among them, sucrose, known as table sugar, is the benchmark compound to measure relative sweetness. It is a disaccharide composed of glucose and fructose, 2 naturally occurring monosaccharides that are themselves sweet-tasting and widely found in fruits, vegetables, and honey (Belloir et al. 2017). The detection threshold for sucrose in humans ranges from 6.8 to 10.2 mM depending on age (Petty et al. 2020). The following disaccharides, maltose, lactose, and trehalose, are also known for their sweet properties. The isomerization of aldo-disaccharides produces sweet ketodisaccharides such as maltulose, isomaltose (palatinose), lactulose, cellobiulose, and melibiulose (Belloir et al. 2017). Inulin, a fructose polymer, and oligofructose, both of which are extracted from chicory roots, are weakly sweet but are widely used in the food industry due to their technological properties (solubility, foam stability, emulsion, fat replacement, etc.) (Franck 2002).

### 2.2 Natural sweeteners

Sugar alcohols (polyols), naturally occurring in fruits and vegetables or produced by the fermentation of microorganisms, such as sorbitol, mannitol, xylitol, erythritol, isomalt, and maltitol offer sweetness with fewer calories than sucrose does (Belloir et al. 2017). D-Allulose (D-psicose), D-tagatose, D-sorbose, and D-allose are rare sugars obtained from microbial bioproduction, and exhibit a sweetness equivalent to 70, 92, 70, and 80% sucrose, respectively (Mooradian et al. 2017). Some amino acids have been reported to have a sweet taste, with decreasing sweetness potency as follows: D-tryptophan (D-Trp), D-histidine (D-His), D-phenylalanine (D-Phe), D-tyrosine (D-Tyr), D-leucine (D-Leu), L-alanine (L-Ala), and glycine (Gly) (Solms 1969). With the exception of L-Ala, these sweet-tasting amino acids correspond to D-enantiomers rather than the L-forms that are typically found in proteins. Many steviol glucosides, such as stevioside, rebaudiosides A, B, C, D, E, and F, dulcoside A, and steviolbioside, have been extracted from stevia (*Stevia rebaudiana*) for their sweet properties. Their sweetness potency is 30 to 240 times higher than those of sucrose (Schiffman and Gatlin 1993; An et al. 2025), but some of them also activate human bitter taste receptors, TAS2Rs (Hellfritsch et al. 2012; Belloir, et al. 2024b), and thus elicit a bitter taste. Moreover, steviosides also possess a licorice off-taste which reduces their acceptability (Prakash et al. 2008). Glycyrrhizin and mogrosides IV and V have been isolated from licorice root and Monk fruit, respectively, and are high-potency sweeteners, but their licorice aftertaste limits their use in the food industry (Schiffman and Gatlin 1993; Lindley 2012). They are currently not allowed in the European Union. Some phenolic compounds extracted from emblic fruit, such as quercetin, methyl gallate, syringic acid, gallic acid, protocatechuic acid, myricetin, kaempferol, naringenin, and rutin, are sweet-tasting compounds, although some of them are known to elicit a pronounced bitterness (Roland et al. 2013). For example, quercetin and syringic acid exhibit detection thresholds of 0.63 and 1.88 mg/mL, respectively (Che et al. 2022), which are lower than that of glucose (≈ 2.39 mg/mL) (Breslin et al. 2021). Interestingly, the activation of TAS1R2/TAS1R3 by 10 saponins (oleanolic acid glycosides) isolated from the *Millettia dubia* De Wild plant was tested. Three of them are characterized by half-maximal effective concentrations (EC_50_) lower than those of sucralose (EC_50_ = 35.8 µM) (Pertuit et al. 2024). Many steroidal and triterpenoid saponins from plants exhibit sweetness (An et al. 2025), but some of them also elicit off-flavors, such as bitterness and/or astringency, which may prevent their use in the food industry (Karolkowski et al. 2023). Finally, compared with the same-purity normal water, heavy water (D_2_O), which is naturally present in small proportions in water, generates a sweet taste in humans (Ben Abu et al. 2021).

### 2.3 Semisynthetic and synthetic sweeteners

Semisynthetic sweeteners are derived from natural molecules that are modified through chemical reactions. They are employed at low concentrations to reduce the caloric content in food and beverages while maintaining sweetness. The most commonly known semisynthetic sweeteners are, with their sweetness potencies, aspartame (160 to 220), sucralose (600), neohesperidin dihydrochalcone (NHDC, 250 to 1,800), perillartine (2,000), alitame (2,000), neotame (11,000), and advantame (20,000) (Laffitte et al. 2016). However, alitame and perillartine remain unauthorized in the European Union.

Synthetic or artificial sweeteners are obtained from chemical synthesis. The sweetness properties of 4 compounds, namely, cyclamate, acesulfame K, saccharin, and lugduname, have been studied (Laffitte et al. 2016). Lugduname is the sweetest compound known and is 230,000 times sweeter than sucrose (Nofre and Tinti 1996), but is not allowed for the human consumption as a sweetener.

Many semisynthetic and synthetic sweeteners act synergistically with each other, which explains why they are very often used in combination. This synergistic effect is particularly useful for the food industry, as it allows for a reduction in the concentration of certain sweeteners that, when used in high doses, can induce undesirable bitter taste. For example, sucralose activates the bitter taste receptors TAS2R1, TAS2R10, and TAS2R46 at millimolar concentrations, whereas a micromolar concentration of sucralose is enough to activate TAS1R2/TAS1R3 (Belloir et al. 2024a). Sucralose has been shown to synergize with acesulfame K, cyclamate, and saccharin (Schiffman and Gatlin 1993). Finally, one alternative for improving the sensory profile of sweeteners is to inhibit the perception of their bitter taste. It has been shown that combining compounds such as saccharin and cyclamate attenuates these undesirable flavors through the mutual inhibition of the bitterness receptors that each activate individually (Behrens et al. 2017). This phenomenon illustrates how sweetener blend formulations can be used to refine taste quality by reducing bitterness while maintaining sweetness intensity.

### 2.4 Sweet-tasting proteins

Only 6 sweet-tasting proteins have been identified from plants: brazzein (6.4 kDa), monellin (11.4 kDa), pentadin (12 kDa), mabinlin (12.4 kDa), thaumatin (22 kDa), and neoculin (previously named curculin, 25 kDa) (Belloir et al. 2017). These proteins can generate an intense sweet taste. For example, thaumatin is approximately 1,600 times sweeter than sucrose on a weight basis and 100,000 times greater on a molar basis (Briand and Salles 2016). Heterologous expression can be an interesting solution for obtaining large quantities of sweet-tasting proteins. The *Pichia pastoris* expression system has been successfully used to produce recombinant proteins such as thaumatin and brazzein (Poirier et al. 2012). Emerging sweet-tasting proteins have also been identified, although not all of them are yet produced on an industrial scale. Honey truffle sweetener has been extracted from Hungarian sweet truffles, and a 20 µg/mL concentration is equivalent to that of a 4% sucrose solution (w/v) (McFarland et al. 2024).

### 2.5 Sweet taste enhancers

Several compounds have been reported as positive allosteric modulators (PAMs) of sweet-tasting compounds, such as SE-2, which enhances the efficacy and potency of sucralose even if it does not elicit a sweet taste on its own. SE-3 and SE-4 also reduce the amount of sugars by up to 50% while preserving the intensity of the perception of sweetness (Zhang et al. 2010). Other PAMs have been shown to increase the sweetness intensity of common sweeteners, including 2,4-dihydroxybenzoic acid, 4-amino-5,6-dimethylthieno[2,3-D]pyrimidin-2(1H)one, and 3-[(4-amino-2,2-dioxido-1H-2,1,3-benzothiadiazin-5-yl)oxy]-2,2-dimethyl-N-propylpropanamide (DuBois and Prakash 2012). These compounds are approved by FEMA (Flavor and Extract Manufacturers Association) as artificial flavors and are named FEMA3798, FEMA4669, and FEMA4701, respectively. In addition, 100 ppm hesperetin (FEMA4313), a flavone identified in oranges, increases the sweetness intensity by 41% in a 5% sucrose solution (Ley et al. 2014). Ribonucleotides can enhance the perception of sweetness when combined with other sweeteners. Synergistic effects have been observed for IMP in blends with sucralose, neotame, or cyclamate and for GMP in blends with sucralose (Belloir et al. 2025). Interestingly, compared with dissolution in normal water, the dissolution of glucose or cyclamate in heavy water enhances the human perception of sweetness (Ben Abu et al. 2021). Moreover, unnatural tripeptides (10 ppm) increase the sweetness intensity of a 5% sucrose solution (Yamada et al. 2019).

### 2.6 Sweet taste inhibitors

A small number of compounds are known to inhibit sweet taste perception. Synthetic sweetness inhibitors include amiloride (Imada et al. 2010), N-(4-cyanophenyl)-N′-[(sodiosulfo)methyl]urea (Muller et al. 1992), zinc sulfate (Keast 2003; Keast et al. 2004), and 2-(2,4-dichlorophenoxy)propionic acid (2,4-DP) (Nakagita et al. 2019) in humans, whereas methyl 4,6-dichloro-4,6-dideoxy-α-D-galactopyranoside, pnitrophenyl α-D-glucopyranoside, chloramphenicol (Jakinovich 1983; Vlahopoulos and Jakinovich 1986a, 1986b), alloxan (Zawalich 1973), iodoacetic acid (Noma and Hiji 1972), zinc chloride, and copper chloride (Iwasaki and Sato 1984; Iwasaki and Sato 1986) have also been reported to inhibit sweetness in animals. Sweet inhibitors from plant-derived triterpenoids, including hodulcin (Kennedy et al. 1988), gymnemic acid (Liu et al. 1992), ziziphin (Meiselman et al. 1976), and lactisole (Sclafani and Pérez 1997; Galindo-Cuspinera et al. 2006), have also been identified. For example, lactisole, extracted from coffee beans, inhibits sweet taste perception in humans but not in rats or mice (Sclafani and Pérez 1997; Galindo-Cuspinera et al. 2006). It is known to suppress the sweetness of acesulfame K, brazzein, cyclamate, D-tryptophan, NHDC, saccharin, sucralose, sucrose, thaumatin, and heavy water (Jiang et al. 2005a; Ben Abu et al. 2021). Lactisole and 2,4-DP have IC_50_ values for aspartame of 65 and 6.2 µM, respectively, indicating that 2,4-DP is more potent than lactisole is (Nakagita et al. 2019). Two sweet taste-suppressing proteins, gurmarin and riboflavin-binding protein, have been detected in *Gymnema sylvestre* and chicken eggs, respectively. Interestingly, gurmarin is a sweetness inhibitor in rodents but not in humans, unlike lactisole, which inhibits the sweet taste only in humans (Sigoillot et al. 2012).

## 3. Umami compounds

**Figure 2:**
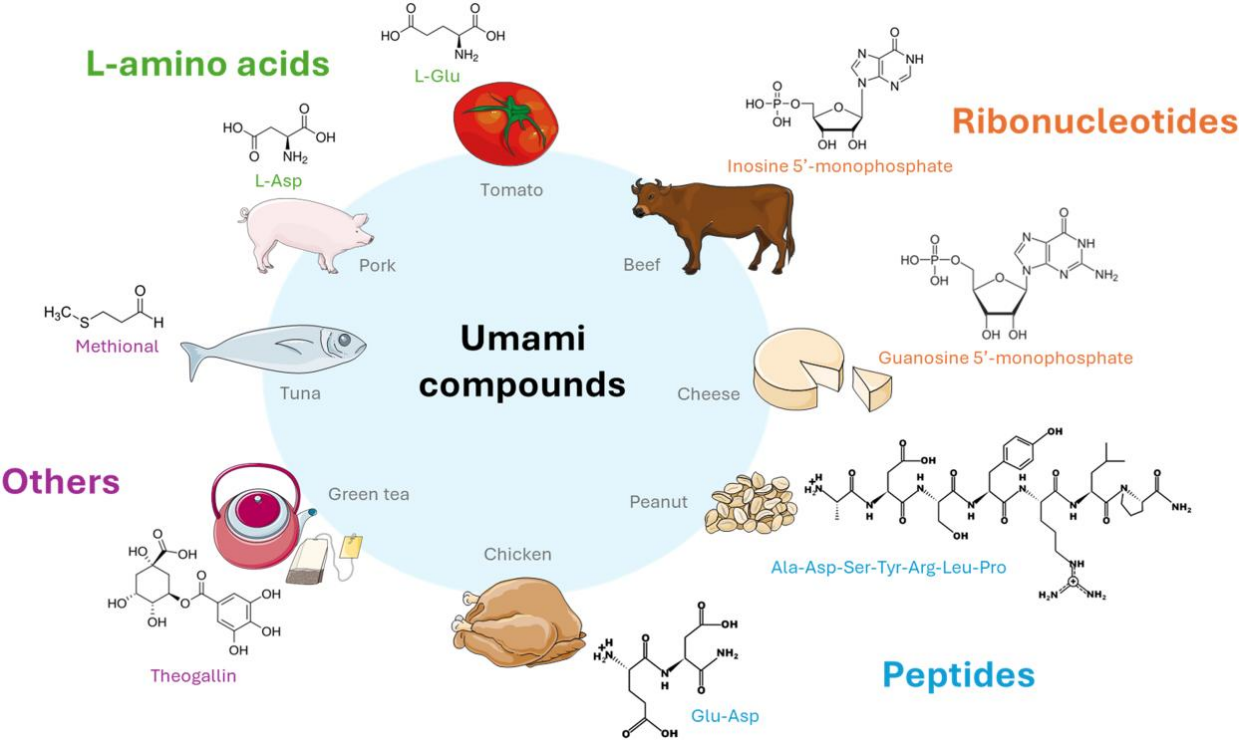
Umami compounds identified in certain food products. L-Glu: L-glutamate; L-Asp: L-aspartate; Ala: L-alanine; Ser: L-serine; Tyr: L-tyrosine; Arg: L-arginine; Leu: L-leucine; Pro: L-proline.

Umami is the characteristic taste generated by monosodium glutamate (MSG), which occurs naturally in cheese, meat, vegetables, mushrooms, and seafood (Ikeda 2002). Other umami tastants have been identified by sensory and/or cellular analysis (Fig. 2).

### 3.1 Amino acids

The 2 amino acids L-Glu and L-Asp elicit an umami taste in humans (Yamaguchi et al. 1971). The human detection threshold for MSG ranges from 0.7 to 3 mM (Roper 2017). In addition, L-Ala, L-serine (L-Ser), Gly, and L-asparagine (L-Asn) weakly activate the human umami taste receptor TAS1R1/TAS1R3, whose weak umami taste has been confirmed by sensory analysis (Toda et al. 2013). L-theanine, identified in green tea, can also increase the umami intensity of MSG (Kaneko et al. 2006).

### 3.2 Nucleotides

Strong synergy between 5′-ribonucleotides, such as IMP and GMP, and MSG is a unique feature of umami (Yamaguchi et al. 1971). Compared with IMP, GMP is more potent, whereas adenosine 5′-monophosphate (AMP) and xanthosine 5′-monophosphate have weaker effects (Li et al. 2002). In pea protein isolates, uridine 5′-monophosphate (UMP) enhances the umami perception of MSG only in combination with AMP (Ongkowijoyo and Peterson 2023). Other nucleotide derivatives formed upon the Maillard reaction of GMP elicit an umami taste and have been observed to synergize with MSG in yeast extracts (Festring and Hofmann 2011). For example, sensory analysis and cellular assay have shown that N^2^-(propylthiomethyl)guanosine 5′-monophosphate, generated by Maillard reactions from GMP, is able to enhance the umami taste of MSG (Suess et al. 2014).

### 3.3 Peptides and derivatives

Many peptides obtained from the hydrolysis of plant and animal proteins or generated synthetically elicit an umami taste or enhance the taste of umami tastants. Umami peptides are primarily short linear peptides (<5 kDa), with dipeptides and tripeptides accounting for the majority of umami peptides identified. They typically contain umami or hydrophilic amino acids (Zhang et al. 2019a). For example, unlike α-Glu-Ala, γ-Glu-Ala, and glutathione (γ-Glu-Cys-Gly) activate TAS1R1/TAS1R3 (Servant and Frerot 2022; Cornut et al. 2025). Glutathione is a typical kokumi substance, which does not elicit a taste by itself but enhances mouthfulness and flavor complexity when combined with basic tastes, including umami (Ohsu et al. 2010). Therefore, kokumi should be distinguished from umami, which is a basic taste providing a a direct savory perception. Interestingly, IMP can modulate the activity of α-Glu-Ala, γ-Glu-Ala, and Glu-Glu-Leu (Servant and Frerot 2022). However, long-chain peptides have different amino acid compositions, with reduced proportions of umami and hydrophilic amino acids. For these peptides, spatial structure may play a more critical role than amino acid composition in determining umami taste (Zhang et al. 2019a). Arg-Gly-Glu-Asn-Glu-Ser-Glu-Glu-Glu-Gly-Ala-Ile-Val-Thr, a tetradecapeptide extracted from peanut protein isolate hydrolysate, has an umami recognition threshold in water of 0.43 mM (Zhang et al. 2019b), which is lower than that of MSG (1.6 mM) (Ikeda 2002). Several peptides obtained from the Maillard reaction have also been identified as umami tastants or enhancers, for example, in Spanish mackerels and peas (Tao et al. 2023; An et al. 2025).

### 3.4 Other compounds

In addition to amino acids and nucleotides, several nonamino acid compounds have been reported to elicit or enhance umami taste. L-lactic acid and succinic acid cause umami taste in beef juice/broth and green tea, respectively (Warendorf et al. 1992; Kaneko et al. 2006). Ibotenic acid activates the umami taste receptor (Servant and Frerot 2022). Gallic acid and theogallin, 2 phenolic compounds identified in green tea, have been shown to synergize with MSG (Kaneko et al. 2006). Several compounds classified by FEMA have been identified as potent umami tastants. For example, FEMA 4267 has a potency of 0.37 µM, whereas that of MSG is 2.31 µM (Servant and Frerot 2022). Sensory analyses have shown that N-cinnamoyl-phenethylamines, such as rubemamine (FEMA 4310) and rubescenamine (FEMA 4773), elicit strong umami taste in water at 50 and 10 ppm, respectively, and modulate the umami taste of MSG (Backes et al. 2015). Naturally occurring pyridines such as 2-hexylpyridine in cooked poultry exhibit an umami taste, but their use in food is limited by their strong odor (Delort et al. 2011). A synthetic analog, FEMA 4832, was developed to have umami properties without an intense odor (Miyazawa et al. 2021). Methional, a volatile compound, enhances the responses of hTAS1R1/hTAS1R3 to various amino acids, including L-Glu and L-Asp (Toda et al. 2018). Menthol analogs have also been identified as umami enhancers at low concentrations by sensory analysis (Backes et al. 2014).

## 4. Structure–function relationships of sweet and umami taste receptors

Sweet and umami taste receptors belong to the class C GPCR family, which features a common structure consisting of a large extracellular domain, called the Venus flytrap domain (VFD), connected to a transmembrane domain (TMD) by a cysteine-rich domain (CRD). Our understanding of the mechanisms leading to the recognition of molecules eliciting the sweet and umami taste modalities has made great strides owing to the structural and functional characterization of TAS1R2/TAS1R3 and TAS1R1/TAS1R3 for the sweet and umami taste receptors, respectively. Structural biology studies have revealed the molecular basis for the detection and modulation of sweet and umami taste molecules. The variable subunit of the 2 receptors determines the agonist profile and, consequently, whether the perceived taste is umami or sweet. By contrast, ligands acting on the shared TAS1R3 subunit modulate taste sensitivity: lactisole raises the detection threshold for umami and sweet compounds, whereas cyclamate lowers it (Xu et al. 2004).

The VFD is organized into 2 lobes, LB1 and LB2, connected by a hinge that allows for the domain to close upon ligand binding, a mechanism reminiscent of a flytrap. Structurally, the VFD closely resembles bacterial periplasmic binding proteins (PBPs) (Quiocho and Higgins 1997), which mediate the transport of small molecules across the periplasmic space (Borrok et al. 2009). This evolutionary convergence has led to the hypothesis that class C GPCRs originate from the fusion of a PBP-like extracellular sensing domain and a 7-transmembrane rhodopsin-like signaling domain. This architecture underscores the evolution of class C receptors, integrating ancient nutrient detection machinery with the signal transduction capabilities of GPCRs. The CRD (≈80 amino acids) typically contains 9 conserved cysteines that form intradomain disulfides, folding them into a domain spanning ≈40 Å that separates the VFD and the TMD (Chun et al. 2012). It has been shown for class C GPCRs that the CRD probably acts as a flexible linker, transmitting conformational changes in the VFD upon ligand binding to the TMD to initiate receptor activation (Chun et al. 2012). The TMD (≈300 amino acids) consists of 7 transmembrane α-helices with short extra/intracellular loops connecting them. Although class C GPCRs exhibit low sequence homology with class A receptors, their TMDs share several conserved structural motifs. The TMD contains allosteric binding sites where small-molecule modulators can bind and modulate receptor activity (Chun et al. 2012). Interestingly, this allosteric pocket in Class C GPCRs appears to spatially overlap with the orthosteric binding site found in Class A GPCRs. Class C GPCRs signal through a sequence of large-scale conformational changes. Ligand binding to the VFD triggers its closure, which is relayed via the CRD to the TMD, leading to G protein activation (Chun et al. 2012). This stepwise mechanism highlights the importance of interdomain communication, although some aspects, particularly in TAS1R1/TAS1R3, are not fully understood.

### 4.1 Structure-function relationships of the sweet taste receptor

**Figure 3:**
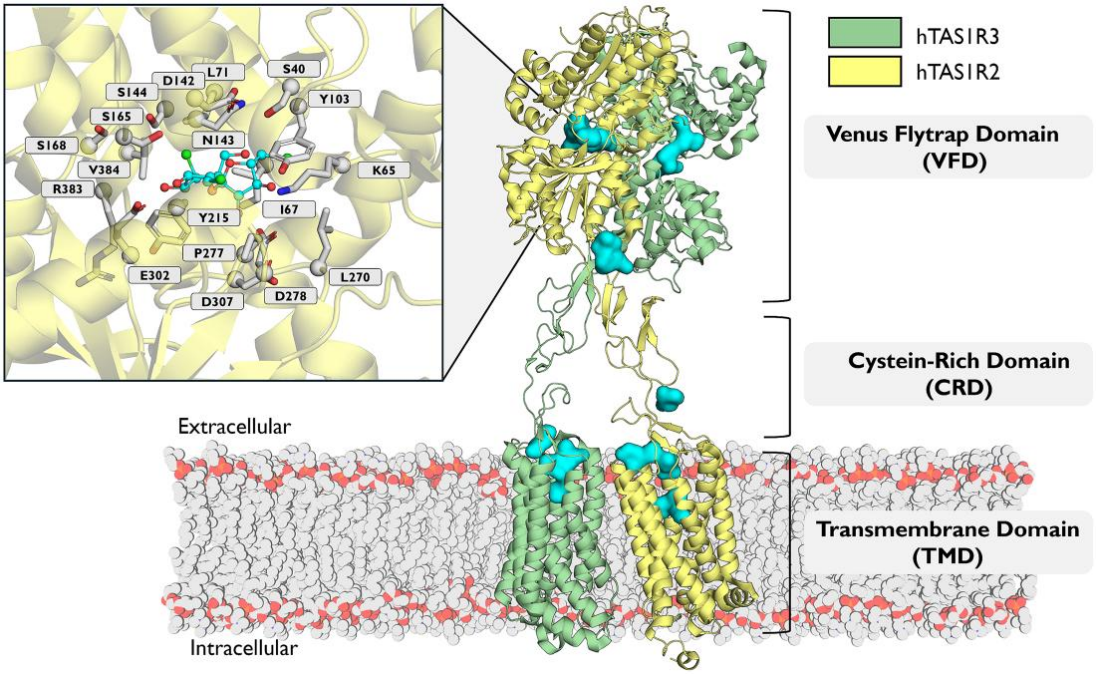
Structural model of the human sweet taste heterodimeric receptor composed of the hTAS1R2 (yellow) and hTAS1R3 (green) subunits. The model was constructed using the available cryo-EM structure (PDB ID: 9NOR) as a template and refined through AlphaFold predictions (Jumper et al. 2021). The receptor comprises 3 main domains: the Venus flytrap domain (VFD), the cysteine-rich domain (CRD), and the transmembrane domain (TMD). Potential ligand-binding pockets identified using the Fpocket algorithm (Le Guilloux et al. 2009) are highlighted in cyan. The inset shows a close-up view of the orthosteric binding site within the VFD of hTAS1R2, with key interacting residues subjected to site-directed mutagenesis (Supplementary Table S1) depicted in gray surrounding the bound sucralose molecule (cyan sticks).

The unique heterodimeric architecture of the sweet taste receptor (Fig. 3), comprising the TAS1R2 and TAS1R3 subunits, has recently been elucidated by cryo-electron microscopy (cryo-EM) (Juen et al. 2025; Shi et al. 2025; Wang et al. 2025). As previously described (Xu et al. 2004; Zhang et al. 2010), sucralose and aspartame, 2 artificial sweeteners, bind to the TAS1R2-VFD binding pocket, which corresponds to the orthosteric binding pocket that also binds most natural sweeteners (Chéron et al. 2019a) (Supplementary Table S1). In addition to the VFD, additional ligand-binding sites have been identified, including allosteric binding sites within the TMDs of both the TAS1R2 (Servant et al. 2011; Cai et al. 2016; Zhao et al. 2018) and TAS1R3 subunits (Jiang et al. 2005a, 2005b; Winnig et al. 2007; Chéron et al. 2019b; Belloir et al. 2025), as well as a binding site in the CRD that accommodates the sweet-tasting protein brazzein (Jiang et al. 2004). This versatility in binding modes explains the wide range of sweet-tasting compounds, despite being mediated by a single receptor.

The canonical activation mechanism, supported by recent cryo-EM structures, involves the binding of orthosteric ligands, inducing a conformational change. This change is characterized by the closure and rotation of the extracellular domains of TAS1R2, with the closure stabilized by electrostatic interactions, such as the K65-D278 salt bridge. Surprisingly, the experimental structures reveal that binding occurs exclusively in the TAS1R2 subunit. These findings support the notion of an auxiliary role for TAS1R3, as proposed earlier (Nuemket et al. 2017), and are in line with asymmetric conformational changes between open and closed forms (Juen et al. 2025), notably with a less pronounced closure and rotation in TAS1R3. The conservation of key binding residues in TAS1R2 across mammals, which differ from those in TAS1R3 (Chéron et al. 2017), supports a shared activation mechanism. However, this mechanism is not universal, as evidenced by species such as cats, which have a nonfunctional *Tas1r2* gene and lack a carbohydrate preference (Jiang et al. 2012). The disulfide bonds in the CRD impart rigidity to its structure, effectively creating a lever-like mechanism that amplifies mechanical constraints (Chéron et al. 2017). This, in turn, enables the efficient transmission of the chemical stimulus from the VFD to the TMD, where it triggers the binding of downstream signaling effectors. The G protein, gustducin, of the sweet taste receptor is likely coupled to cytosolic part of TAS1R2-TMD, as suggested by a model derived from the cryo-EM structure of the receptor combined with site-directed mutagenesis experiments (Shi et al. 2025). Although the structure of the sweet taste receptor has been solved, it remains unclear whether G-protein coupling occurs via the TAS1R2 or the TAS1R3 subunit, a question that also extends to umami receptor signaling. Nevertheless, numerous experimental and structural clues support a cis-activation model, in which the same subunit is responsible for both ligand recognition and subsequent G-protein coupling, suggesting a direct and coordinated mechanism of signal transduction within a single monomer. Cryo-EM structures have so far captured only a subset of ligand classes and conformational states, so there is still uncertainty about when TAS1R3 directly binds sweeteners in its own VFD or TMD pocket versus when it functions primarily as an allosteric transmitter and G protein-coupling partner. These observations contrast with studies that demonstrated that the VFD of both subunits bind natural sugars (sucrose and glucose) and sucralose (Nie et al. 2005; Maîtrepierre et al. 2012). Since cryo-EM directly visualizes bound ligands in a subset of states, if the TAS1R3 pocket is low-occupancy, flexible, or only used by some ligands, it may not appear as a well-defined ligand density.

The sweet taste receptor is also expressed in tissues outside the mouth, although it is unclear whether its activation mechanism is the same in these tissues (Laffitte et al. 2014). The new experimental structures focus on the TAS1R2/TAS1R3 heterodimer, but they do not address the structure of the TAS1R3/TAS1R3 homodimer. A number of studies indicate the TAS1R3 homodimer as functional sweet taste receptor (Zhao et al. 2003; Zopun et al. 2018; Dubovski et al. 2023). In nontaste tissues (e.g. adipocytes), TAS1R3 expression in the absence of TAS1R2 is common, and functional studies show “sweet-responsive” signaling through a presumed TAS1R3 homomer, albeit with low affinity for physiological sugars and often unclear endogenous ligands (Zhao et al. 2003; Masubuchi et al. 2017). Some reports also show TAS1R3 alone mediating calcium taste or metabolic responses, again supporting a homomeric receptor with distinct pharmacology from the canonical heterodimer (Tordoff et al. 2012). These observations support that the TAS1R3 subunit is able to couple to the G protein.

### 4.2 Structure–function relationships of the umami taste receptor

**Figure 4:**
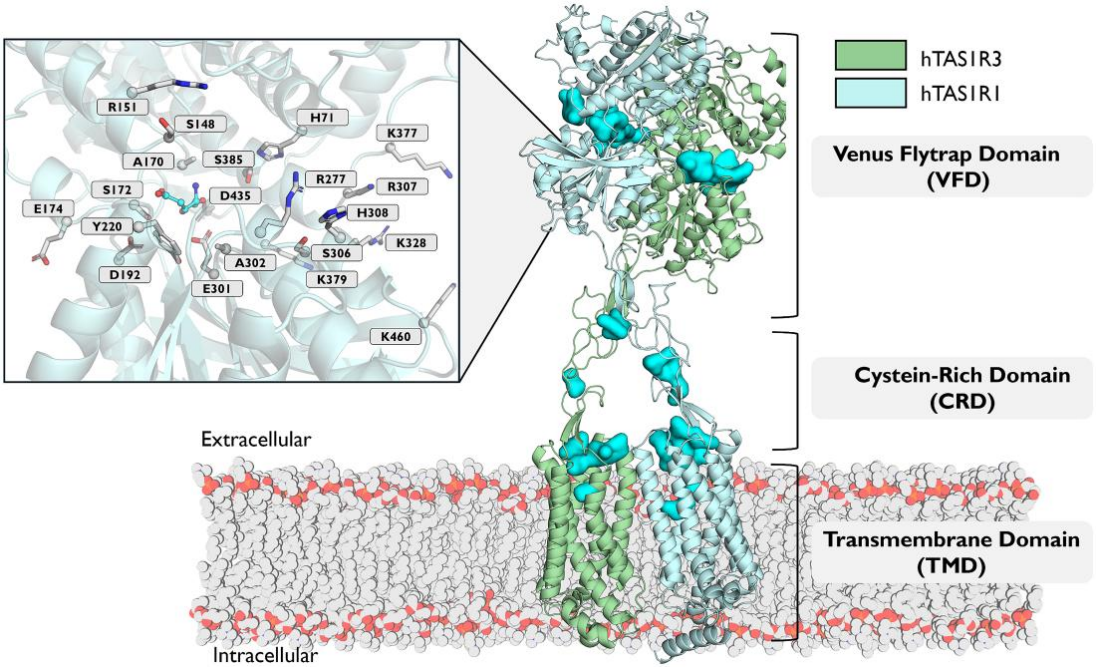
Structural model of the human umami taste heterodimeric receptor composed of the hTAS1R1 (blue) and hTAS1R3 (green) subunits. The model was constructed using AlphaFold predictions (Jumper et al. 2021). The receptor comprises 3 main domains: the Venus flytrap domain (VFD), the cysteine-rich domain (CRD), and the transmembrane domain (TMD). Potential ligand-binding pockets identified using the Fpocket algorithm (Le Guilloux et al. 2009) are highlighted in cyan. The inset shows a close-up view of the orthosteric binding site within the VFD of hTAS1R1, with key interacting residues subjected to site-directed mutagenesis (Supplementary Table S2) depicted in gray surrounding the bound L-Glu (L-glutamate) molecule (cyan sticks). Molecular docking was performed using AutoDock Vina (v1.2.5) (Eberhardt et al. 2021). Proteins and ligands were prepared via the MGLTools Python script package. Docking grids were designed to contain all the residues identified by fpocket binding pocket detection software. Exhaustiveness was set to 50, and the number of random seeds was set to 123. All the other parameters were kept at their default values.

In vertebrates, the umami taste is primarily mediated by a heterodimer composed of taste receptor type 1 (TAS1R1) and taste receptor type 3 (TAS1R3) (Li et al. 2002; Nelson et al. 2002). The umami taste receptor is expressed primarily in the taste bud cells of the tongue and palate (Yoshida and Ninomiya 2016), where it plays a key role in detecting L-amino acids. TAS1R1 and TAS1R3 subunits must form a heterodimer to fold properly, traffic, and initiate signaling, as has been observed for the TAS1R2/TAS1R3 receptor and the GABA_B_ receptor (GABA_B1_/GABA_B2_) (Zhao et al. 2003). Although both subunits contribute to the overall architecture of the receptor, only the TAS1R1 subunit should be responsible for agonist binding and activation of the associated G protein (Fig. 4).

To date, the full-length structure of the TAS1R1/TAS1R3 umami taste receptor has not been experimentally resolved. However, structural insights have been gained from related systems. The VFD of the medaka fish TAS1R2a, a teleost paralog of human TAS1R2 has been elucidated (Nishihara et al. 2024; Policarpo et al. 2024) and provides a valuable structural template for understanding ligand recognition in class C VFDs (Oike et al. 2007; Nuemket et al. 2017; Ishida et al. 2024). More recently, the cryo-EM structure of hTAS1R2/TAS1R3 was determined at 2.8 Å resolution (Juen et al. 2025), and the expected modular organization of a class C GPCR, including the VFD, CRD, and TMD, was confirmed. Although this structure concerns the sweet taste receptor, the high sequence and functional similarity suggest that TAS1R1/TAS1R3 probably adopts a comparable architecture.

These recent structural advances offer a strong foundation for developing a more detailed understanding of the structure–function relationships that govern ligand binding, receptor activation, and signal transduction in the umami taste pathway. Umami taste receptors, like other class C GPCRs, feature a distinctive large N-terminal extracellular region known as the VFD, which is essential for ligand binding and activation. In fish, distinct loop–loop contacts at the VFD interface stabilize the dimers of TAS1R2a/TAS1R3 (Nuemket et al. 2017). Beyond the disulfide bond, hydrophobic, and electrostatic interactions at the VFD interfaces strengthen the heterodimer (Ray and Hauschild 2000).

TAS1R1/TAS1R3 is widely conserved across vertebrate species, reflecting its fundamental role in nutrient detection (Toda et al. 2021; Wolsan and Sato 2022). Despite this evolutionary conservation, species-specific structural variations have emerged, particularly in the amino acid sequences of the ligand-binding domains. These sequence modifications can modulate ligand recognition and specificity (Raliou et al. 2009). For example, in most mammals, umami receptors respond broadly to a variety of L-amino acids. In contrast, hTAS1R1/TAS1R3 shows a marked preference for L-Glu, attributed to a group of residues forming the ligand-binding cavity (Toda et al. 2013). On more divergent evolutionary paths, some species have lost umami receptor function or adapted it to new sensory roles. Domestic cats, for example, possess a variant of TAS1R1 with mutations that eliminate L-Glu binding (McGrane et al. 2023). Conversely, chimera studies in hummingbirds suggest that sequence changes in the VFD can retune ligand preference: swapping VFD amino acids between chicken and hummingbird TAS1R1/TAS1R3 shifts receptor responses between amino acids and sugars. This reflects their unique dietary dependence on flower nectar (Baldwin et al. 2014). More generally, these examples illustrate the plasticity of TAS1R receptors under ecological pressures. Receptor tuning can shift with a few substitutions (Cockburn et al. 2022; Cramer et al. 2022) or with broader dietary transitions over evolution (Toda et al. 2021), and in some lineages, the umami receptor can even be reduced or lost (Wolsan and Sato 2022).

L-Glu is the primary agonist of the TAS1R1/TAS1R3 receptor, and its binding interactions have been extensively characterized through site-directed mutagenesis, in vitro functional assays, and computational modeling (Diepeveen et al. 2022). These studies have identified the hinge region of TAS1R1-VFD as the main binding site (Supplementary Table S2). Within this pocket, L-Glu is stabilized by a combination of ionic interactions, hydrogen bonds, and van der Waals forces. Key residues such as S172, D192, Y220, and E301 form a network of interactions that coordinate with the amino and carboxylate groups of L-Glu in a manner that closely resembles the ligand recognition mechanism observed in mGluRs. Similarly, very similar amino acids, such as L-Asp, activate the receptor albeit less potently than L-Glu does. Other amino acids do not generally strongly activate hTAS1R1/hTAS1R3, but for some species, the TAS1R1 pocket is broadly tuned to recognize a range of amino acids in rodents (Nelson et al. 2002).

Synergism in umami is well documented. The synergy between L-Glu and certain 5′-ribonucleotides, such as IMP and GMP, has been extensively investigated. The molecular basis for these synergisms lies in a secondary binding site on the receptor. Chimeric, mutagenesis, and modeling studies revealed that IMP and GMP bind to a distinct site of TAS1R1-VFD adjacent to the L-Glu pocket. Specifically, mutation of residues H71, R277, S306, and H308 in TAS1R1 abolished the in vitro enhancing effect of IMP without preventing L-Glu binding (Zhang et al. 2008). These findings confirmed that an independent IMP-specific pocket was located toward the outer opening of the VFD. L-Glu and IMP seem to overlap slightly; IMP binding alone does not close the VFD, but it stabilizes the VFD-L-Glu bound in the closed-active conformation.

The TAS1R3 subunit, while originally thought to function as a coreceptor, also has binding capacity in its own VFD and TMD. Recent findings have shown that the TAS1R3 subunit may also participate in ligand binding, especially for nucleotides and other modulators (Diepeveen et al. 2022). Interestingly, IMP can also interact with TAS1R3-VFD, and this interaction modulates signaling. Moreover, compared with TAS1R1, TAS1R3-VFD can bind directly to some umami ligands but with a lower affinity (Belloir et al. 2025). The involvement of TAS1R3 complexifies the 2-site model. In cats, which lack L-Glu detection (McGrane et al. 2023) and rely on nucleotide detection for umami taste, these findings suggest that changes in feline TAS1R1 prevent L-Glu binding but that TAS1R3 may have compensatory effects, allowing for nucleotides to trigger the receptor on their own. This implies that the nucleotide binding site (whether on TAS1R1 or TAS1R3) can in some cases produce an active receptor state on its own. This finding also strongly suggests a cross-talk between umami and sweet taste modalities since TAS1R3 is present in both receptors (Cornut et al. 2025). The complex seems to feature multiple potential ligand binding sites: the orthosteric site in TAS1R1-VFD for amino acids, an adjacent site at the periphery or interface of the 2 VFDs for 5′-ribonucleotides, and additional sites in TAS1R3-VFD or -TMD for other modulators. By analogy with other class C receptors, CRDs may also contain potential binding sites. These different sites allow for the recognition of a wide variety of agonists. Each subunit of TAS1R1/TAS1R3 may contribute to ligand binding.

## 5. Extraoral expression

Sweet and umami taste receptors are found not only in the oral cavity but also in numerous tissues and organs, including the gut, pancreas, bladder, and brain. In the gut, TAS1Rs are expressed throughout the gastrointestinal (GI) tract, notably in chemosensory and enteroendocrine cells in rodents and humans (Behrens and Lang 2022). Recent review articles describe the main known functions of these taste receptors in the GI tract; thus, the reader should refer to them for this specific topic (Kochem 2017; Behrens and Lang 2022). Briefly, the stimulation of enteroendocrine cells by sweet or umami compounds modulates the release of peptide hormones involved in metabolic regulation (for example, glucagon-like peptide 1 (GLP-1) (Jang et al. 2007) or ghrelin (Vancleef et al. 2015)), whereas the stimulation of chemosensory cells by umami-like compounds seems to induce defense responses in the organism (Howitt et al. 2020). Recently, it has been hypothesized that the sweet taste receptor in the GI tract could be involved in the development of allergic diseases (Xian et al. 2025).

TAS1R2 and TAS1R3 are also expressed in the brain, and are abundantly expressed in the hypothalamus, which plays a central role in feeding behaviors and energy homeostasis (Ren et al. 2009; Herrera Moro Chao et al. 2016; Benford et al. 2017; Kohno 2017; Lazutkaite et al. 2017). The stimulation of the sweet receptor in the brain induces an anorexigenic effect (Kohno 2017), and the expression of TAS1R3 in the hypothalamus is decreased in diet-induced obese mice and *ob/ob* mice (Herrera Moro Chao et al. 2016). Compared with the TAS1R3 subunit, the TAS1R1 subunit is also expressed at lower levels in the brain (Ren et al. 2009; Voigt et al. 2015).

The TAS1R3 subunit has been found in the human liver (Taniguchi 2004), and all 3 TAS1R subunits have been found in pancreatic β-cells in rodents and humans. The stimulation of the sweet receptor in this organ induces insulin secretion not only via a mechanism involving glucose but also a mechanism involving fructose, in synergy with glucose (Nakagawa et al. 2009; Kyriazis et al. 2012). Stimulation of the umami receptor in pancreatic β-cells regulates insulin secretion (Oya et al. 2011).

Interestingly, the TAS1R subunits are also found in parts of the body that are not directly involved in the detection or assimilation of nutrients. In chemosensory cells of the human upper airway, the TAS1R2/TAS1R3 receptor is expressed in colocalization with TAS2R bitter taste receptors (Lee et al. 2014), and activation of the sweet taste receptor inhibits the TAS2R-induced defensive response via an alternate G protein different from α-gustducin. A hypothesis to explain this phenomenon is that the regular level of glucose in the airway surface liquid is sufficient to inhibit TAS2R release of antimicrobial peptides under normal conditions. However, during infection, the glucose concentration decreases when the bacteria are used, resulting in the inhibition of TAS2R activity. This response is specific to upper airway cells because it is not observed in human bronchial epithelial cells of the lower airway (Lee et al. 2014; Carey and Lee 2019).

TAS1R subunits have also been observed in the bladder and urethra in rodents and humans (Elliott et al. 2011; Schmidt et al. 2024). The stimulation of urethral chemosensory cells by sweet compounds is possibly involved in protection against urinary tract infection by increasing micturition via a TAS1R3-dependent pathway (a TAS1R3-independent pathway was also highlighted) (Schmidt et al. 2024). Urethral chemosensory cells also respond to the umami compound MSG, certainly to facilitate the detection of bacteria in the urethral lumen (Deckmann et al. 2014).

All the TAS1R subunits have been found in blood leukocytes in humans, with TAS1R3 having the highest expression of the 3 subunits (Malki et al. 2015; Ball et al. 2023). The heterodimerization of TAS1R3 with mGluR2 in these cells is involved in MSG-induced immune responses (Ball et al. 2023). The dimerization of TAS1R3 with TAS1R2 is involved in the chemotactic migration of polymorphonuclear leukocytes toward the sweet compound saccharin (Malki et al. 2015).

**Figure 5:**
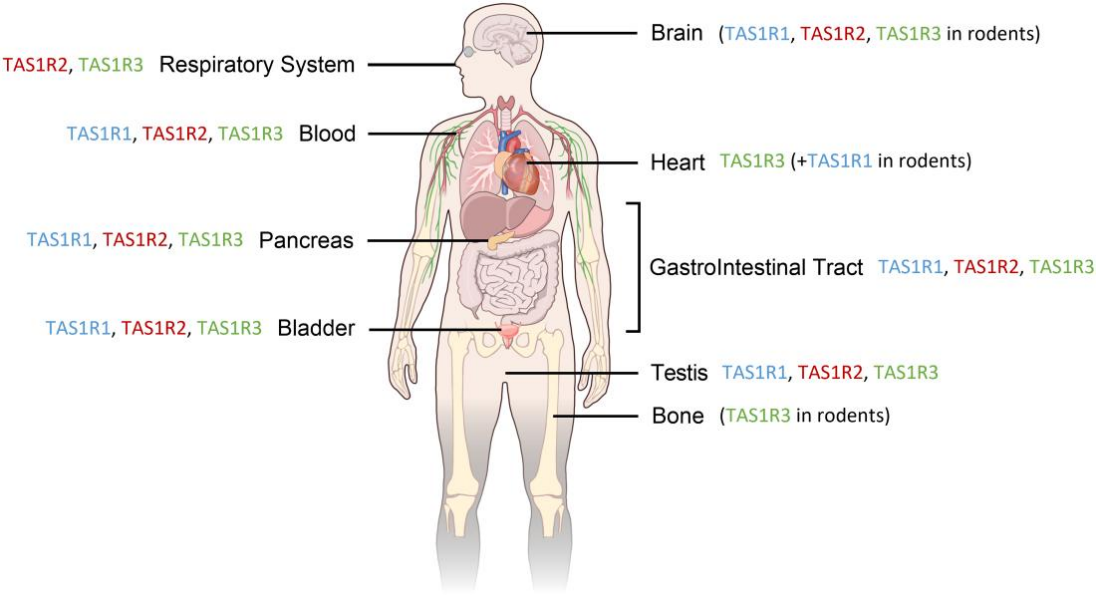
Extraoral expression of TAS1R subunits. The expression of TAS1R1, TAS1R2, and TAS1R3 was characterized in human and rodent tissues using immunohistochemistry and/or molecular biology techniques (in parentheses: the expression was analyzed (brain, bone) or found (heart) only in rodent tissues).

In the rodent heart, TAS1R1 and TAS1R3 are expressed, whereas in the human heart, only TAS1R3 is expressed (Foster et al. 2013). TAS1R3, but not TAS1R1 and TAS1R2, has also been found in the bone tissue of mice and is involved in osteoclastogenesis (Yoshimura et al. 2025). All the TAS1R subunits are expressed in the testis, and the umami receptor is expressed in spermatozoa in rodents and humans (Li 2013). In most cases, the physiological role of these receptors remains to be elucidated. A synthesis of the extraoral expression of TAS1R receptors is presented in Fig. 5.

## 6. Conclusion and perspectives

Over the past 2 decades, remarkable advances in structural biology, molecular pharmacology, and comparative genomics have fundamentally reshaped our understanding of sweet and umami perceptions TAS1R2/TAS1R3 and TAS1R1/TAS1R3 heterodimers have emerged as multisite GPCRs capable of detecting a vast diversity of natural and synthetic ligands. Their modular organization, with orthosteric, allosteric, and cooperative binding sites, provides a molecular basis for the sensitivity, selectivity, and synergism that characterize sweet and umami tastes. In particular, the L-Glu-IMP synergy in umami perception exemplifies the cooperative allostery that defines class C GPCR function.

The recent resolution of cryo-EM structures of the TAS1R2/TAS1R3 sweet taste receptor has offered important insights into the molecular architecture and activation mechanisms of this receptor, revealing the asymmetric roles of the TAS1R subunits and their coupling to gustducin. Although the structure of the TAS1R1/TAS1R3 umami taste receptor remains unresolved, its high sequence and functional similarity to those of TAS1R2/TAS1R3 provide a solid framework for modeling ligand binding and conformational transitions. Future high-resolution structures of TAS1R1/TAS1R3 will be essential for elucidating the molecular determinants of amino acid and nucleotide binding, and the precise mechanisms underlying synergistic activation.

Beyond the oral cavity, the discovery of TAS1R expression in multiple extraoral tissues, including the gut, pancreas, airways, brain, and bone, has revealed a broader physiological significance for these receptors as nutrient sensors involved in metabolic and immune regulation. Their roles in processes such as GLP-1 secretion, mucociliary clearance, and bone remodeling highlight the integration of chemosensory signaling with systemic homeostasis. However, in most tissues, the downstream signaling pathways and physiological role of TAS1R activation remain poorly defined and constitute a promising frontier for future research.

From an evolutionary perspective, comparative analyses across vertebrates have shown remarkable plasticity in TAS1R receptor function, where minor sequence variations can redirect ligand specificity from amino acids in humans to nucleotides in cats or sugars in hummingbirds. This evolutionary adaptability underscores how taste receptor diversification has shaped feeding behaviors and ecological niches.

In the future, the integration of cryo-EM, molecular dynamics simulations, and in vivo functional studies will be essential for establishing a dynamic and mechanistic understanding of TAS1R signaling. Such knowledge could guide the rational design of taste modulators, novel sweeteners, and umami enhancers, as well as therapeutic agents targeting TAS1R-mediated pathways in metabolism and immunity. Ultimately, elucidating how TAS1R receptors decode chemical signals at both the sensory level and the systemic level will deepen our understanding of the connections between taste, nutrition, and health.

## Supplementary Material

bjag010_Supplementary_Data

## Data Availability

No new data were generated or analyzed in support of this research.
