# Supplementary material for: Sweet and umami TAS1R receptors: from molecular recognition to physiological function
Source: Chem Senses. 2026 Mar 19;51:bjag010. doi: 10.1093/chemse/bjag010 (PMC13034538; doi:10.1093/chemse/bjag010)
Supplement: bjag010_Supplementary_Data [file bjag010_supplementary_data.docx]

**Sweet and Umami TAS1R Receptors: From Molecular Recognition to Physiological Function – Supplementary**

**﻿**

**Clémence Cornut^1^, Christine Belloir^1^, Adeline Karolkowski^1^, Maxence Lalis^2^_,_ Sandrine Chometton^1^, Sébastien Fiorucci^2^, Jérémie Topin^2,*^ and Loïc Briand^1,*^**

^1^ Université Bourgogne Europe, Institut Agro, CNRS, INRAE, UMR CSGA, 21000 Dijon, France

^2^ Institut de Chimie de Nice UMR7272, Université Côte d’Azur, CNRS, 28 Avenue Valrose, 06108 Nice, France

**Table S1.** Summary of hTAS1R2 mutations (for the sweet taste receptor).

| id | Pos | Reference |
| --- | --- | --- |
| 1 | S40 | (Maillet et al. 2015) |
| 2 | Y103 |  |
| 3 | D142 |  |
| 4 | S144 |  |
| 5 | S165 |  |
| 6 | S168 |  |
| 7 | Y215 |  |
| 8 | D278 |  |
| 9 | E302 |  |
| 10 | D307 |  |
| 11 | R383 |  |
| 12 | I67 | (Sanematsu et al. 2025) |
| 13 | Y103 | (Masuda et al. 2012) |
| 14 | D142 |  |
| 15 | S144 |  |
| 16 | S165 |  |
| 17 | P277 |  |
| 18 | D278 |  |
| 19 | E302 |  |
| 20 | D307 |  |
| 21 | E382 |  |
| 22 | R383 |  |
| 23 | I67 | (Shi et al. 2025) |
| 24 | L71 |  |
| 25 | Y103 |  |
| 26 | D142 |  |
| 27 | N143 |  |
| 28 | S165 |  |
| 29 | P277 |  |
| 30 | D278 |  |
| 31 | E302 |  |
| 32 | C384 |  |
| 33 | S40 | (Zhang et al. 2010) |
| 34 | Y103 |  |
| 35 | D142 |  |
| 36 | P277 |  |
| 37 | D278 |  |
| 38 | E302 |  |
| 39 | R383 |  |
| 40 | K65 |  |
| 41 | L279 |  |
| 42 | D307 |  |
| 50 | S40 | (Liu et al. 2011) |
| 51 | D142 |  |
| 52 | S144 | (Xu et al. 2004) |
| 53 | E302 |  |
| 54 | D278 | (Laffitte et al. 2022) |
| 55 | E382 |  |

**Table S2.** Summary of hTAS1R1 mutations (for the umami taste receptor).

| id | Position | Reference |
| --- | --- | --- |
| 1 | H71 | (Zhang et al. 2008) |
| 2 | R277 |  |
| 3 | S306 |  |
| 4 | H308 |  |
| 5 | S172 |  |
| 6 | D192 |  |
| 7 | Y220 |  |
| 8 | E301 |  |
| 9 | S385 |  |
| 10 | S148 | (Toda et al. 2013) |
| 11 | R151 |  |
| 12 | A170 |  |
| 13 | E174 |  |
| 14 | A302 |  |
| 15 | D435 |  |
| 16 | R307 |  |
| 17 | K377 |  |
| 18 | K379 |  |
| 19 | K460 |  |
| 20 | M320 |  |
| 21 | K328 |  |
| 22 | S172 |  |
| 23 | E301 |  |
| 24 | S172 | (Narukawa et al. 2014) |
| 25 | E301 |  |
| 26 | A110 |  |

**References:**

Laffitte A, Belloir C, Neiers F, Briand L. 2022. Functional Characterization of the Venus Flytrap Domain of the Human TAS1R2 Sweet Taste Receptor. Int J Mol Sci. 23(16):9216. <https://doi.org/10.3390/ijms23169216>

Liu B et al. 2011. Molecular Mechanism of Species-Dependent Sweet Taste toward Artificial Sweeteners. J Neurosci. 31(30):11070–11076. <https://doi.org/10.1523/JNEUROSCI.0791-11.2011>

Masuda K et al. 2012. Characterization of the Modes of Binding between Human Sweet Taste Receptor and Low-Molecular-Weight Sweet Compounds. PLOS ONE. 7(4):e35380. <https://doi.org/10.1371/journal.pone.0035380>

Narukawa M et al. 2014. l-Theanine elicits umami taste via the T1R1 + T1R3 umami taste receptor. Amino Acids. 46(6):1583–1587. <https://doi.org/10.1007/s00726-014-1713-3>
